# Temporal Changes in Antimicrobial Resistance, SpvB Polyproline Linker Length, and Pulsed-Field Gel Electrophoresis Types of *Salmonella* Gallinarum from Chickens in South Korea: A 25-Year Retrospective Study (2002–2026)

**DOI:** 10.3390/vetsci13101089

**Published:** 2026-10-14

**Authors:** O-Mi Lee, So-Hee Lee, Hye-Rin Lee, Yeong Ju Yu, Min Beom Kim, Sung-Il Kang, Myeong Ju Chae, Moon Her, Jin Hyun Kim

**Affiliations:** 1 Avian Disease Division, Animal and Plant Quarantine Agency, 177, Hyeoksin 8-ro, Gimcheon-si 39660, Gyeongsangbuk-do, Republic of Korea; lomi78@korea.kr (O.-M.L.); tdj04051@gmail.com (S.-H.L.); leehr4612@gmail.com (H.-R.L.); yeongju9213@gmail.com (Y.J.Y.); minbum5935@korea.kr (M.B.K.); ksilion@korea.kr (S.-I.K.); herm@korea.kr (M.H.); 2 Animal Disease Diagnostic Division, Animal and Plant Quarantine Agency, 177, Hyeoksin 8-ro, Gimcheon-si 39660, Gyeongsangbuk-do, Republic of Korea; chaemj@korea.kr

**Keywords:** *Salmonella* Gallinarum, fowl typhoid, antimicrobial resistance, multidrug resistance, SpvB, polyproline linker, pulsed-field gel electrophoresis, pulsotype, molecular epidemiology

## Abstract

Fowl typhoid, caused by *Salmonella* Gallinarum (SG), remains an important poultry disease. We characterized 136 SG isolates, one per farm, from diagnostic submissions of diseased chickens to the national veterinary laboratory in South Korea from 2002 to 2026, comparing two periods (2002–2010 and 2011–2026). Antimicrobial susceptibility was determined by broth microdilution, the *spvB* polyproline linker (PPL; the region joining the two functional domains of the SpvB virulence factor) was sequenced, and genetic relatedness was assessed by *Xba*I–pulsed-field gel electrophoresis (PFGE). Resistance to nalidixic acid was most frequent (93.4%), followed by streptomycin (83.1%) and ciprofloxacin (71.3%). Resistance to ampicillin, tetracycline, and nalidixic acid was less frequent in 2011–2026 (false discovery rate-adjusted *p* < 0.05), and these associations persisted after adjustment for chicken type. Multidrug resistance was detected in 52.2% of isolates, without significant change between periods. However, 13 of the 34 multidrug-resistant isolates from 2002–2010 were resistant to four or more antimicrobial classes, whereas all 37 from 2011–2026 were resistant to exactly three classes. Five PPL lengths of 13–21 prolines were identified, and their distribution differed between periods (*p* = 0.016). Isolates carrying ≥17 prolines were more frequent in 2011–2026 than in 2002–2010 (*p* < 0.01). PFGE yielded 61 pulsotypes in 27 clusters, with two dominant clusters detected across years and chicken types. These findings indicate that the PFGE-defined SG population showed temporal phenotypic changes within Korean poultry flocks, underscoring the need for continued surveillance of resistance and genotype.

## Simple Summary

Fowl typhoid is a severe bacterial disease in chickens caused by *Salmonella* Gallinarum. In South Korea, it has been controlled through vaccination, improved farm hygiene, and antibiotic treatment, and the number of cases has declined markedly in recent years. We examined 136 isolates collected from diseased chickens over a 25-year period (2002–2026) to determine how they have changed. Resistance to several long-established antibiotics was lower in the more recent group of strains, and resistance patterns were simpler, but approximately half of the strains remain resistant to three or more classes of antibiotics. SpvB is a bacterial protein that contributes to disease, and its two parts are joined by a short chain of the amino acid proline, called the polyproline linker. Longer polyproline linker variants were more frequent in the later study period. Molecular typing by pulsed-field gel electrophoresis showed that a small number of closely related strains were detected in Korean farms during most of the study period and across different chicken types. These findings describe how *Salmonella* Gallinarum changed as the disease declined, provide a reference for detecting changes in the strains if outbreaks increase again, and can inform the choice of antibiotics by veterinarians treating the disease.

## 1. Introduction

Fowl typhoid (FT) is an acute or chronic septicemic disease of chickens caused by *Salmonella enterica* subsp. *enterica* serovar Gallinarum biovar Gallinarum (SG) and remains an important cause of economic losses in poultry production because of its high morbidity and mortality in affected flocks [1,2]. Together with pullorum disease, FT has been largely eradicated from commercial poultry in most developed countries through test-and-slaughter programs and strict biosecurity, but it remains endemic and economically important across much of Asia, Africa, and South America [1,2,3]. Because SG is host-restricted and undergoes extensive genome degradation, it forms a genetically homogeneous, highly clonal population, a feature that shapes both its epidemiology and the interpretation of molecular-typing data [3].

In Korea, FT was first reported in 1992 and subsequently became a major bacterial disease of poultry. Following the introduction of nationwide SG9R vaccination in 2001, outbreak numbers peaked in 2002 and then declined substantially as vaccine use increased [4]. Outbreaks have since occurred only sporadically rather than as large-scale epidemics [5], and clinical FT has become increasingly uncommon in Korean poultry in recent years. The collection of isolates analyzed in the present study (2002–2026) encompasses the period following the peak of FT outbreaks and the subsequent decline in disease occurrence in Korea, providing an opportunity to investigate long-term changes in the molecular epidemiology of SG. Despite the marked reduction in disease incidence, concerns remain regarding the persistence and transmission of SG within poultry production systems. In particular, the poultry red mite (*Dermanyssus gallinae*), which is widespread in commercial layer farms, has been implicated as a potential reservoir and vector of SG. In Korea, identical or closely related genotypes have been recovered from mites, dust, and dead birds on the same farm [6], and experimental studies have demonstrated that poultry red mites are capable of transmitting SG between chickens [7]. Another emerging epidemiological concern is the increasing difficulty in distinguishing vaccine-like and field-associated strains. Field isolates genetically related to the SG9R vaccine strain have been reported in several countries [8], and recent investigations in Korea have identified SG9R-like field isolates that possess molecular and phenotypic characteristics associated with wild-type SG [5]. These findings highlight the need for continued surveillance and molecular characterization of circulating SG populations.

Antimicrobials continue to be used for the treatment and management of FT outbreaks, and β-lactams, aminoglycosides, and fluoroquinolones have been commonly administered in affected poultry flocks in Korea and elsewhere [3,9]. Sustained antimicrobial use has been associated with the emergence and persistence of resistant SG isolates and an increasing prevalence of multidrug-resistant (MDR) isolates over the past two decades [9,10]. This trend is consistent with global observations; in the largest genomic dataset currently available, 40.4% of SG isolates exhibited MDR phenotypes and carried acquired antimicrobial-resistance genes [11]. Reduced susceptibility to fluoroquinolones in Korean SG has been linked to mutations in the quinolone resistance-determining region (QRDR) of *gyrA*, particularly at codons Ser83 and Asp87 [12]. More recent ciprofloxacin (CIP)-resistant isolates have likewise been associated with *gyrA* mutations, frequently in combination with MDR [13]. Nevertheless, comprehensive long-term assessments of temporal changes in antimicrobial-resistance phenotypes among Korean SG isolates remain limited.

The virulence of SG is strongly influenced by the plasmid-borne *spv* (*Salmonella* plasmid virulence) operon, which comprises the regulatory gene *spvR* and the structural genes *spvABCD* and is required for systemic infection in animal hosts [14,15]. The *spvB* gene encodes SpvB, a mono(ADP-ribosyl)transferase that modifies actin at Arg177 and disrupts the host actin cytoskeleton, a function that contributes to intracellular proliferation and virulence [15,16,17,18]. Structurally, SpvB consists of a C-terminal ADP-ribosyltransferase domain connected to an N-terminal domain through a polyproline linker (PPL) [16]. Deletion of *spvB* markedly attenuates virulence in chickens [19]. The PPL exhibits length polymorphism among SG isolates, and previous studies have suggested that variation in PPL length may be associated with virulence-related phenotypes and may assist in differentiating field isolates from SG9R-related strains [20,21]. However, despite their potential biological significance, the long-term distribution of PPL lengths and their temporal dynamics in Korean SG populations remain poorly understood.

Pulsed-field gel electrophoresis (PFGE) has historically been one of the most widely used methods for assessing genetic relatedness among *Salmonella* isolates and remains useful for epidemiological comparison with historical datasets, despite its limited discriminatory power for highly clonal organisms [22,23,24]. Earlier Korean studies consistently demonstrated low genetic diversity among SG isolates, revealing only a small number of *Xba*I-PFGE profiles and no clear association between genotype and chicken breed or geographic origin [4,10]. A similar clonal population structure has been reported internationally, including phylogenomic evidence of clonal diversification among SG isolates from Brazil and highly conserved genomes among outbreak-associated strains from Colombia [25,26,27]. Previous Korean studies of SG have each covered a limited period [4,9,10,20], and changes in resistance phenotypes, PPL length, and PFGE types across the transition from high FT incidence to its decline have not been assessed within a single, consistently tested collection. Accordingly, this study investigated antimicrobial resistance, SpvB PPL length, and *Xba*I-PFGE genotypes among 136 SG isolates recovered from chickens in Korea between 2002 and 2026. The study aimed to evaluate temporal changes in antimicrobial resistance and PPL length and to characterize the PFGE-defined population structure of SG isolates in Korea.

## 2. Materials and Methods

### 2.1. Bacterial Isolates

A total of 136 SG isolates were recovered from postmortem tissues collected from chickens submitted to the Avian Disease Division of the Animal and Plant Quarantine Agency, Gyeongsangbuk-do, South Korea, for the diagnosis of suspected FT between 2002 and 2026. All isolates originated from diagnostic submissions of diseased or dead chickens, and none were obtained through active or routine surveillance sampling. One representative isolate was included per farm to avoid overrepresentation of farms from which multiple isolates were recovered during the same outbreak investigation. The distribution of the 136 isolates by year of isolation and chicken type is summarized in Table 1. Layers were the most common chicken type (70 isolates, 51.5%), followed by broilers (29, 21.3%), Korean native chickens (16, 11.8%), broiler breeders (12, 8.8%), and Baeksemi (9, 6.6%). No SG was isolated between 2021 and 2023, and a single isolate was recovered in each of 2024, 2025, and 2026.

For temporal comparison, the isolates were grouped into two periods, 2002–2010 (*n* = 62) and 2011–2026 (*n* = 74). The cut-point was defined before the statistical comparisons on the basis of national FT outbreak data. The annual mean number of FT outbreaks reported to the Korea Animal Health Integrated System (KAHIS, https://home.kahis.go.kr, accessed on 30 March 2026) was 82.9 in 2002–2010 and 27.2 in 2011–2025, calculated as the total number of reported outbreaks divided by the number of years in each interval, and 2026 was excluded because annual data were incomplete. Therefore, the two periods correspond to phases of higher and lower FT occurrence in Korea and also yielded groups of similar size. Because the periods differ in duration (9 and 16 years) and annual isolate numbers were uneven, the comparison was intended to describe differences between two broad phases rather than a continuous longitudinal trend. As the isolates analyzed here originated from diagnostic submissions, their number does not directly reflect national incidence, and it may also have been influenced by changes in surveillance intensity or submission practices.

Bacterial isolation and identification were performed as follows. At necropsy, internal organs showing gross lesions suggestive of FT, mainly the liver and spleen, were aseptically collected. A small portion of each organ was streaked directly onto MacConkey agar (Difco Laboratories, Detroit, MI, USA), and the plates were incubated for 24 h at 37 °C. Presumptive colonies were further identified by biochemical testing using the Vitek 2 system (bioMérieux, Lyon, France). All confirmed *Salmonella* isolates were serotyped according to the Kauffmann–White scheme using commercially available O and H antisera (BD Difco, Sparks, MD, USA). Because conventional serotyping does not discriminate SG from *Salmonella* Pullorum, biovar differentiation was performed by polymerase chain reaction (PCR), as described previously [28]. All isolates were stored in tryptic soy broth (Difco) supplemented with 20% glycerol at −80 °C.

### 2.2. Antimicrobial Susceptibility Testing

Antimicrobial susceptibility testing was performed using the broth microdilution method with the Sensititre™ CMV3AGNF plate (Trek Diagnostic Systems, Cleveland, OH, USA), according to the manufacturer’s instructions. Minimum inhibitory concentrations (MICs) were determined for the following antimicrobials (with tested concentration ranges): amoxicillin/clavulanic acid (AUG, 1/0.5–32/16 μg/mL), ampicillin (AMP, 1–32 μg/mL), azithromycin (AZI, 0.12–16 μg/mL), cefoxitin (FOX, 0.5–32 μg/mL), ceftiofur (XNL, 0.12–8 μg/mL), ceftriaxone (CRO, 0.25–64 μg/mL), chloramphenicol (CHL, 2–32 μg/mL), ciprofloxacin (CIP, 0.015–4 μg/mL), gentamicin (GEN, 0.25–16 μg/mL), nalidixic acid (NAL, 0.5–32 μg/mL), sulfisoxazole (FIS, 16–256 μg/mL), streptomycin (STR, 2–64 μg/mL), tetracycline (TET, 4–32 μg/mL), and trimethoprim/sulfamethoxazole (SXT, 0.12/2.38–4/76 μg/mL). Because the highest FIS concentration on the CMV3AGNF plate (256 μg/mL) is lower than the Clinical and Laboratory Standards Institute (CLSI) resistance breakpoint of 512 μg/mL, FIS MICs were additionally determined by broth microdilution over an extended range of 16–512 μg/mL in accordance with CLSI document M07 (12th ed., 2024) [29]. The MICs obtained from the extended assay were used for the interpretation of FIS susceptibility. MICs were interpreted according to CLSI document M100 (33rd ed., 2023) [30], except for STR, XNL, and AZI, for which the breakpoints of the National Antimicrobial Resistance Monitoring System (NARMS) were applied [31]. CLSI M100 provides no breakpoints for STR or XNL and provides an AZI breakpoint only for *Salmonella* Typhi, and the NARMS resistance monitoring criteria were therefore used for these three agents to allow comparison with other surveillance data (STR ≥ 32 μg/mL, the criterion used by NARMS since 2014, XNL ≥ 8 μg/mL, and AZI ≥ 32 μg/mL). A single set of interpretive criteria was applied retrospectively to all isolates irrespective of the year of isolation, so that the temporal comparisons would not be affected by breakpoint revisions during the study period. The interpretive source for each agent is given in Table 2 and Table S2. *Escherichia coli* ATCC 25922 and *Pseudomonas aeruginosa* ATCC 27853 were used as quality-control strains. MDR was defined as resistance to at least one agent in three or more antimicrobial classes, following the numerical criterion proposed by Magiorakos et al. [32]. The proposal was developed for Enterobacteriaceae other than *Salmonella* and *Shigella*, and it was therefore used here for the numerical definition of MDR only. The 14 agents tested were assigned to the 10 antimicrobial classes shown in Table 2 and Table S2, namely aminoglycosides (GEN, STR), aminopenicillins (AMP), β-lactam/β-lactamase inhibitor combinations (AUG), cephamycins (FOX), third-generation cephalosporins (XNL and CRO), quinolones (CIP and NAL), folate pathway inhibitors (FIS and SXT), phenicols (CHL), tetracyclines (TET), and macrolides (AZI). Agents assigned to the same class were counted once, so that resistance to both CIP and NAL, to both GEN and STR, or to both FIS and SXT was counted as resistance to a single class.

### 2.3. Pulsed-Field Gel Electrophoresis

PFGE was performed with the restriction endonuclease *Xba*I (Invitrogen, Carlsbad, CA, USA) according to the PulseNet protocol of the Centers for Disease Control and Prevention [23]. *Salmonella* serotype Braenderup H9812 restricted with *Xba*I was used as the universal size standard for gel normalization. PFGE band profiles were analyzed using BioNumerics software (version 8.0; Applied Maths, Sint-Martens-Latem, Belgium), and genetic similarity was calculated using the Dice coefficient, with clustering performed by the unweighted pair group method with arithmetic mean. Band position tolerance and optimization were set at 1.5% and 0.5%, respectively. For descriptive purposes, clusters were defined at ≥90% similarity, and isolates with indistinguishable PFGE patterns were assigned to the same pulsotype, in accordance with established criteria for the interpretation of PFGE patterns [22]. PFGE was performed once for each isolate, and runs with poor band resolution were repeated. Because SG is highly clonal and *Xba*I-PFGE has limited discriminatory power in this serovar, PFGE clusters were used to describe relatedness among isolates and were not interpreted as evidence of recent common ancestry or of specific transmission events.

### 2.4. Determination of Polyproline Linker Length in the spvB Gene

The PPL length in the *spvB* gene was determined for all 136 SG isolates. The region of *spvB* encoding the PPL, which connects the N- and C-terminal domains of SpvB, was amplified by PCR using primers targeting the proline-repeat-encoding region, which were designed on the basis of a previous report [21], yielding expected amplicons of approximately 610 bp, with the exact size varying with PPL length. PCR products were purified and sequenced in both directions by Sanger sequencing at Macrogen (Seoul, Republic of Korea). Forward and reverse chromatograms were inspected manually for base-calling quality and assembled into a consensus sequence before translation, and ambiguous reads were resequenced. PPL length was operationally defined as the number of consecutive proline residues in the translated linker region of SpvB, as described previously [21]. Comparison of the PPL-region sequences of all 136 isolates identified five unique sequences, corresponding to the five PPL lengths observed. These sequences have been deposited in GenBank under accession numbers QB081781–QB081785.

### 2.5. Statistical Analysis

Antimicrobial resistance rates for each agent and the prevalence of MDR were compared between the two study periods (2002–2010 and 2011–2026) using Pearson’s chi-square test without continuity correction when all expected cell counts were five or greater, and Fisher’s exact test when any expected cell count was less than five. Because 10 agents with at least one resistant isolate were compared, the *p* values for these comparisons were additionally adjusted using the Benjamini–Hochberg false discovery rate procedure, and both nominal and adjusted values are reported. The distribution of SpvB PPL lengths between the two periods was compared using the Fisher–Freeman–Halton exact test, which was regarded as the primary test for PPL length. In an additional exploratory (post hoc) analysis, isolates were dichotomized at ≥17 prolines, that is, at lengths longer than the predominant 15-proline variant, because the two distributions diverged at this point. This comparison was performed by Fisher’s exact test, and the conditional maximum likelihood odds ratio (OR) with its exact 95% confidence interval (CI) was calculated. The chicken-type composition of the two periods was compared using the Fisher–Freeman–Halton exact test. To assess whether the period associations were explained by differences in chicken-type composition, logistic regression models including period and chicken type (five categories, with layers as the reference category) were fitted for the outcomes that differed significantly between periods (AMP, TET, and NAL resistance and ≥17 prolines). Because several cells contained zero counts, which causes complete separation in conventional maximum likelihood estimation, Firth’s penalized likelihood method was used, with profile penalized likelihood CIs and penalized likelihood ratio tests. Sensitivity analyses were performed by moving the cut-point at each year boundary from 2008/2009 to 2013/2014 and by excluding the three isolates recovered in 2024–2026, using Fisher’s exact test. All tests were two-sided, and *p* values < 0.05 were considered statistically significant. Analyses were performed using R version 4.6.1 (R Foundation for Statistical Computing, Vienna, Austria). The stats package was used for the chi-square tests, Fisher’s exact tests, and *p* value adjustment, and the logistf package (version 1.26.1) was used for Firth’s logistic regression.

### 2.6. Use of Generative Artificial Intelligence

Generative artificial intelligence tools were used only to assist with English language editing of text written by the authors. ChatGPT Business (OpenAI, GPT-5.6 Sol) was used during the preparation and revision of the manuscript.

## 3. Results

### 3.1. Antimicrobial Susceptibility and Temporal Comparison of Resistance

**Figure 1:**
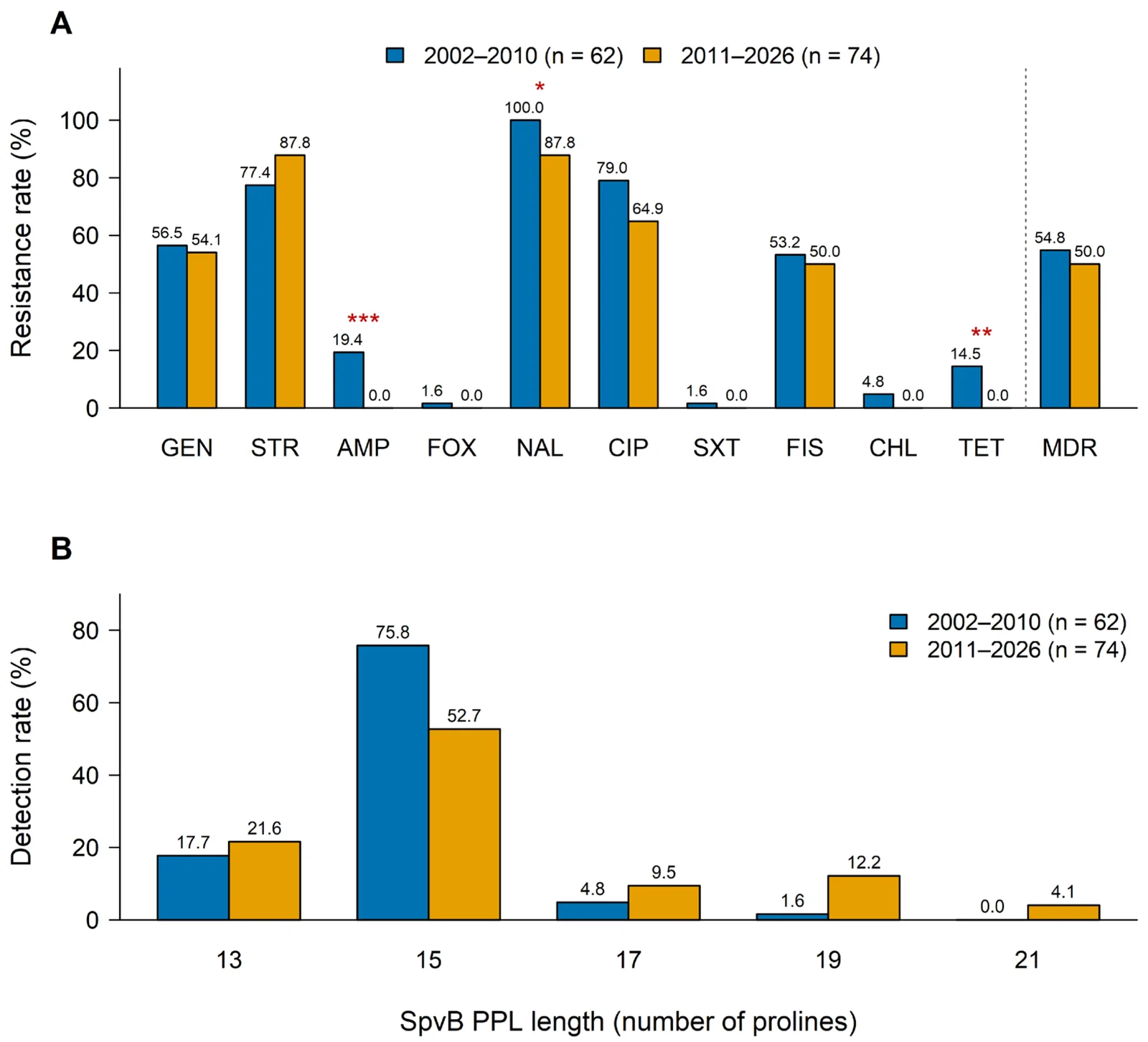
Antimicrobial resistance and distribution of SpvB PPL lengths among *Salmonella* Gallinarum isolates recovered from diseased chickens in South Korea, by study period. (**A**) Percentage of isolates resistant to each antimicrobial agent with at least one resistant isolate and to three or more antimicrobial classes (MDR). The value above each bar is the percentage of resistant isolates in that period. Asterisks indicate Benjamini–Hochberg-adjusted *p* values for the period comparison (* *p* < 0.05, ** *p* < 0.01, and *** *p* < 0.001). (**B**) Distribution of SpvB PPL lengths. The value above each bar is the percentage of isolates with that PPL length in that period. AMP, ampicillin; CHL, chloramphenicol; CIP, ciprofloxacin; FIS, sulfisoxazole; FOX, cefoxitin; GEN, gentamicin; MDR, multidrug resistance; NAL, nalidixic acid; PPL, polyproline linker; STR, streptomycin; SXT, trimethoprim/sulfamethoxazole; TET, tetracycline.

The antimicrobial resistance profiles and MIC distributions of the 136 SG isolates are shown in Table 2 and Figure 1A. Overall, resistance was most frequent to NAL (93.4%), STR (83.1%), and CIP (71.3%), followed by GEN (55.1%) and FIS (51.5%). Resistance to AMP (8.8%), TET (6.6%), CHL (2.2%), FOX (0.7%), and SXT (0.7%) was low, and no isolate was resistant to AUG, CRO, XNL, or AZI. Although resistance to CIP was frequent, the MIC_50_ and MIC_90_ values were 1 and 2 μg/mL, respectively, indicating that the MICs of most resistant isolates were at or one dilution above the resistance breakpoint. Resistance to AMP, TET, and NAL was significantly less frequent in 2011–2026 than in 2002–2010 (*p* < 0.01, and Benjamini–Hochberg adjusted *p* ≤ 0.013), whereas resistance rates to the remaining agents did not differ between the two periods (*p* > 0.05). The MDR proportion was lower in 2011–2026 (50.0%) than in 2002–2010 (54.8%), but the difference was not significant (*p* > 0.05).

Because the chicken-type composition differed between the two periods (*p* < 0.001), with broilers and broiler breeders more common in 2002–2010 and layers, Korean native chickens, and Baeksemi more common in 2011–2026 (Table 1), the period comparisons were adjusted for chicken type. After adjustment, the lower odds of resistance in 2011–2026 remained significant for AMP (adjusted OR [aOR] 0.03, 95% CI 0.0003–0.28), TET (aOR 0.05, 95% CI 0.0003–0.42), and NAL (aOR 0.07, 95% CI 0.0005–0.59), although the estimates were imprecise because of sparse cells (Table S3). In the sensitivity analyses, the direction of these associations was unchanged for all alternative cut-points and after exclusion of the 2024–2026 isolates (Table S3).

### 3.2. Antimicrobial Resistance Patterns

The antimicrobial resistance patterns of the 136 isolates are presented in Table 3. A total of 22 resistance patterns were identified. The most common pattern was CIP-GEN-NAL-STR-FIS (25.0%), followed by CIP-NAL-STR (17.6%). Resistance to four or more antimicrobial classes was observed in 13 isolates, all of which were recovered during 2002–2010, and no such isolate was detected in 2011–2026. These 13 isolates accounted for 38.2% of the 34 MDR isolates from that period, whereas all 37 MDR isolates from 2011–2026 were resistant to exactly three classes. In contrast, isolates resistant to a single antimicrobial agent were more frequent in 2011–2026 (nine isolates, 12.2%) than in 2002–2010 (one isolate, 1.6%), and the only fully susceptible isolate was detected in 2011–2026. These findings suggest a shift from complex MDR phenotypes toward simpler resistance profiles in 2011–2026.

### 3.3. PPL Length Variation in SpvB

The distribution of PPL lengths among the 136 isolates is shown in Table 4 and Figure 1B. Five PPL lengths ranging from 13 to 21 prolines were identified. A PPL length of 15 prolines was the most common (63.2%), followed by 13 prolines (19.9%), whereas 17, 19, and 21 prolines were less frequent (7.4%, 7.4%, and 2.2%, respectively). The distribution of PPL lengths differed between the two periods (*p* = 0.0161). The predominance of 15 prolines was less pronounced in 2011–2026 (52.7%) than in 2002–2010 (75.8%), whereas the proportion of isolates carrying 17 or more prolines was higher in 2011–2026 (25.7%) than in 2002–2010 (6.5%) (exploratory analysis, *p* = 0.0028, OR 4.96, 95% CI 1.52–21.29). This association remained after adjustment for chicken type (aOR 4.56, 95% CI 1.58–15.79) and with the alternative cut-points (Table S3). The longest variant, 21 prolines, was detected only in 2011–2026 (4.1%, in isolates from 2017 and 2018).

### 3.4. PFGE Cluster Analysis

**Figure 2:**
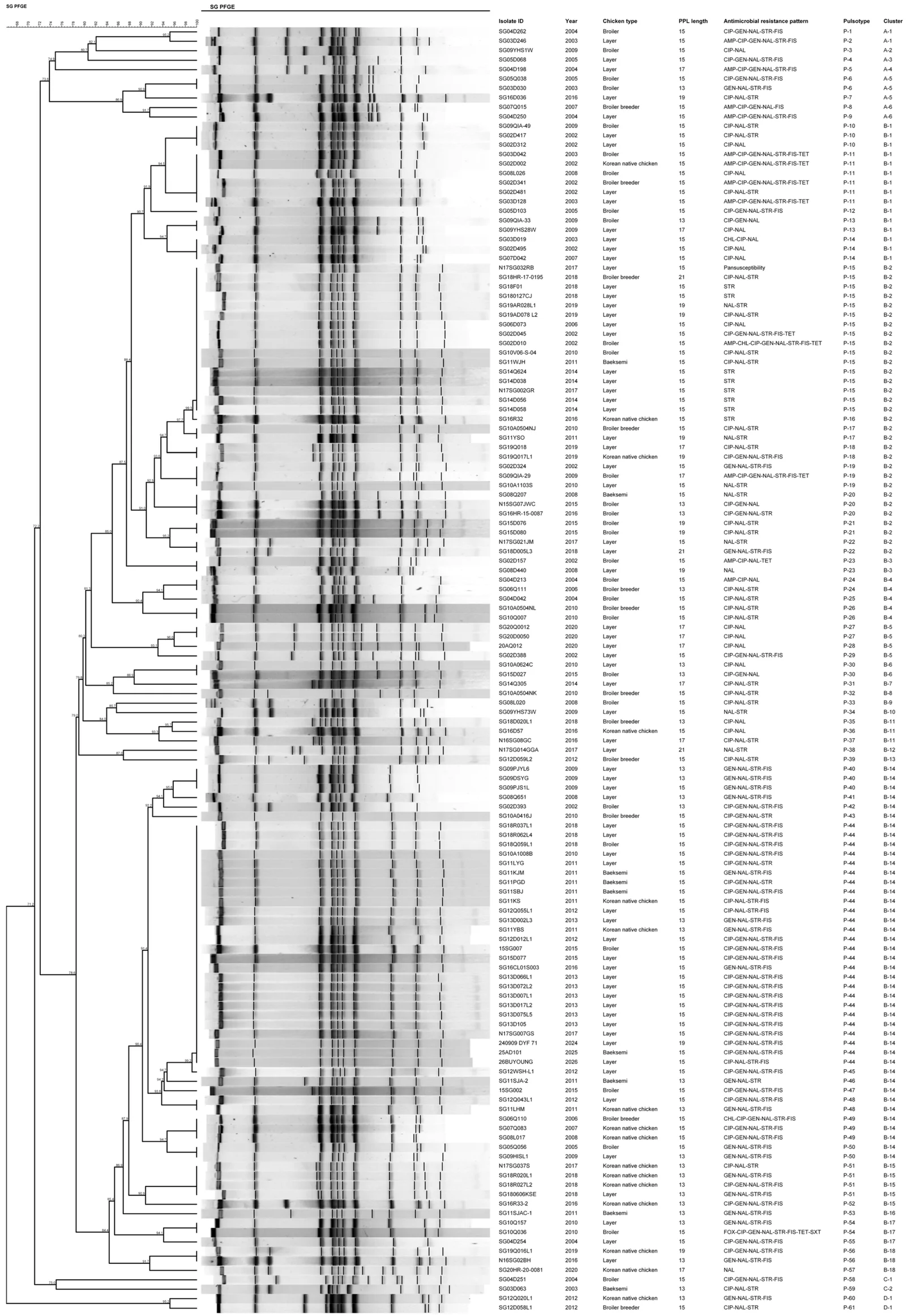
Dendrogram of *Xba*I–pulsed-field gel electrophoresis patterns of *Salmonella* Gallinarum isolates recovered from diseased chickens in South Korea between 2002 and 2026. Clustering was performed by the unweighted pair group method with arithmetic mean based on the Dice coefficient, and clusters were defined at a 90% similarity threshold. Cluster designations are indicated on the right, and the isolation year, chicken type, antimicrobial resistance pattern, and polyproline linker length are shown for each isolate. *Salmonella* serotype Braenderup H9812 was used as the size standard for gel normalization. Pansusceptibility indicates susceptibility to all antimicrobial agents tested. AMP, ampicillin; FOX, cefoxitin; CHL, chloramphenicol; CIP, ciprofloxacin; GEN, gentamicin; NAL, nalidixic acid; STR, streptomycin; FIS, sulfisoxazole; TET, tetracycline; SXT, trimethoprim/sulfamethoxazole.

The dendrogram of the *Xba*I-PFGE patterns among the 136 isolates is presented in Figure 2. *Xba*I-PFGE analysis of the 136 isolates yielded 61 pulsotypes, which were grouped into 27 PFGE clusters at a 90% similarity threshold. B-14 (30.9%) and B-2 (22.8%) were the predominant clusters, jointly representing 53.7% of the isolates, followed by B-1 (11.0%). The remaining isolates were distributed among 24 minor clusters comprising one to five isolates each. Cluster B-14 included isolates recovered from 2002 to 2026 and B-2 from 2002 to 2019, whereas B-1 was detected only from 2002 to 2009. B-14 and B-2 each included isolates from all five chicken types, namely layers, broilers, broiler breeders, Korean native chickens, and Baeksemi (Table S4). Baeksemi is a crossbred chicken produced by mating male meat-type breeders with female commercial layers. Phenotypic characteristics differed between the major clusters. MDR was frequent in B-14 (38 of 42 isolates, 90.5%) but uncommon in B-2 (6 of 31, 19.4%), and all eight isolates resistant to STR alone belonged to B-2 and were recovered between 2014 and 2018 (Table S1 and Table S4). Isolates carrying ≥17 prolines were most frequent in B-2 (10 of 31, 32.3%) and in the 24 minor clusters combined (11 of 48, 22.9%), and were rare in B-14 (1 of 42, 2.4%) and B-1 (1 of 15, 6.7%) (Table S4). Identical or closely related pulsotypes were repeatedly detected across different years and chicken types, indicating repeated detection of genetically similar isolates over the study period. Geographic origin was not consistently recorded for the historical submissions, and the association between PFGE clusters and region could therefore not be assessed.

## 4. Discussion

In the present study, we characterized the antimicrobial resistance, SpvB PPL length, and PFGE population structure of 136 SG isolates recovered from chickens in Korea over a 25-year period. Resistance to AMP, TET, and NAL was lower and resistance profiles were simpler in 2011–2026, and these associations remained after adjustment for chicken type. The proportion of isolates carrying 17 or more prolines was higher in 2011–2026, and two dominant PFGE clusters were detected across years and chicken types during most of the study period.

The overall resistance profile of the isolates was dominated by NAL, STR, and CIP. This pattern is consistent with earlier reports on Korean SG isolates collected between 2002 and 2007 [9] and between 2013 and 2018 [10], in which quinolone and aminoglycoside resistance were also predominant. Notably, all isolates in the present study remained susceptible to AUG, CRO, XNL, and AZI. Extended-spectrum cephalosporin resistance has been reported in *Salmonella* isolates from breeder hatcheries and food animals in China [33,34], whereas no such resistance was detected in this diagnostic SG collection. This may reflect limited use of these agents for the treatment of FT in Korea or the absence of the relevant resistance genes from this host-restricted serovar, but neither possibility was examined in the present study. Continued monitoring remains warranted because the acquisition of extended-spectrum β-lactamase genes would considerably narrow the therapeutic options available for outbreak control.

NAL resistance was the most frequent phenotype throughout the study period. In previous studies, fluoroquinolone resistance in Korean SG has been attributed primarily to point mutations in the QRDR of *gyrA*, and plasmid-mediated quinolone resistance (PMQR) genes have also been detected in domestic isolates [12,13]. In the present study, CIP resistance was frequent, but the MIC_50_ and MIC_90_ values were 1 and 2 μg/mL, respectively, indicating that the MICs of most resistant isolates were at or one dilution above the resistance breakpoint. MICs in this range exceed the CLSI ciprofloxacin breakpoint for *Salmonella*, which was lowered from ≥4 to ≥1 μg/mL because of treatment failures in human extraintestinal infections [35], but their relevance to the treatment of FT in poultry has not been established. Isolates carrying double *gyrA* mutations have been reported to show considerably higher MICs [13]. On the basis of these earlier findings, the low-level resistance observed here may reflect a single *gyrA* substitution or the presence of PMQR determinants, and the high prevalence of NAL resistance may indicate that target-site mutations are common in this population. However, QRDR sequencing and PMQR screening were not performed in the present study, and these mechanisms could not be confirmed for the isolates analyzed here. Further investigation of QRDR mutations and PMQR genes is therefore needed to define the relative contribution of each mechanism and to monitor the emergence of high-level fluoroquinolone resistance in Korean SG.

The significantly lower frequency of AMP, TET, and NAL resistance in 2011–2026 coincided with the changing epidemiology of the disease. Continuous use of the SG9R live vaccine in Korea for more than two decades [5], together with improved biosecurity, has been accompanied by a marked decline in FT, and no SG was isolated in the present study between 2021 and 2023. One hypothesis is that fewer outbreaks required less therapeutic intervention, so that β-lactams, tetracyclines, and fluoroquinolones, long used for the treatment of FT in Korea [9], exerted less selective pressure. However, antimicrobial use data were not analyzed in this study, and the decline may equally reflect changes in disease incidence, vaccination, biosecurity, the PFGE-defined population structure, or other factors. The three agents nonetheless followed different trajectories. AMP and TET resistance was not detected in 2011–2026, whereas NAL resistance remained widespread, at 87.8% in 2011–2026 compared with 100% in 2002–2010. If the chromosomal target-site mutations reported previously in Korean SG [12,13] underlie the NAL resistance observed here, they would be stably inherited and expected to persist in a clonally circulating, host-restricted serovar even as antimicrobial use declines. Given the progressive restriction of enrofloxacin use in poultry [36,37], whether quinolone susceptibility eventually recovers will only become apparent through continued surveillance.

The proportion of MDR isolates was 52.2% overall, with no significant difference between the two periods (54.8% in 2002–2010 and 50.0% in 2011–2026). Comparable or higher prevalences have been reported in earlier Korean studies. Among 105 isolates collected between 2002 and 2007, 63.8% were classified as MDR [9], whereas all 30 isolates recovered from farms with FT outbreaks between 2013 and 2018 exhibited an MDR phenotype [10]. MDR has therefore remained a consistent feature of Korean SG for more than two decades, and the differences in reported prevalence are likely to reflect variation in the number of isolates, the antimicrobial panels tested, the grouping of agents into classes, and the sampling context rather than a genuine temporal trend. In the present collection, for example, counting CIP and NAL as separate classes would have raised the MDR prevalence to 76.5%. Nevertheless, the composition of resistance profiles changed appreciably in the present study. Isolates resistant to four or more antimicrobial classes were confined to 2002–2010, and all 37 MDR isolates recovered in 2011–2026 were resistant to exactly three classes. Taken together, these observations, along with the increase in isolates resistant to a single agent in 2011–2026, suggest a shift from complex to simpler resistance phenotypes, a change that would not be apparent from MDR prevalence alone. Reduced antimicrobial selective pressure is one possible explanation for this shift, but all eight isolates resistant to STR alone belonged to cluster B-2, which was more frequent in 2011–2026, suggesting that changes in the relative representation of PFGE-defined lineages may also have contributed. Although no isolate resistant to four or more classes was detected in 2011–2026, isolates with such complex resistance profiles may still occur at low frequency and cannot be considered eliminated.

The distribution of PPL lengths differed between the two periods, with a lower proportion of the predominant 15-proline variant and a higher proportion of variants carrying 17 or more prolines in 2011–2026. The PPL connects the N- and C-terminal domains of SpvB, and its length has been proposed to modulate the ADP-ribosyltransferase activity of the protein toward actin [15,16,17,18]. Deletion of *spvB* attenuates SG in chickens [19], and Korean isolates have been reported to carry an extended PPL relative to other *Salmonella* serovars, with subgroups differentiated in part by PPL length [21]. This length variation was first reported by Kwon and Cho [38], who found nine prolines in the SG9R vaccine strain and a longer tract in virulent field isolates, although the functional consequence of the additional prolines was not determined. A temporal increase in PPL length has also been reported among Korean isolates recovered between 2014 and 2018, but the absolute lengths differed from those observed here, most likely because of differences in the delineation of the linker region [20]. Our findings extend these observations over a longer period, and the 21-proline variant was detected only in 2011–2026 in this collection (in isolates from 2017 and 2018). Because the ≥17-proline threshold was chosen after inspection of the data, this comparison should be regarded as exploratory. No functional or infection experiments were performed in the present study, and PPL length is only one of several determinants of SG pathogenicity. Any link between the longer PPL variants and increased virulence therefore remains a hypothesis that requires experimental testing.

*Xba*I-PFGE analysis revealed a largely clonal population, with two dominant clusters together accounting for more than half of the isolates. Both clusters included isolates from multiple chicken types and from widely separated years, and identical or closely related pulsotypes were repeatedly detected during most of the study period. A comparable pattern was reported for Korean isolates collected between 2013 and 2018, in which a limited number of PFGE types predominated, and identical types were shared between farms belonging to the same or different integrated poultry companies [10]. Similar clonal structures have been described for outbreak isolates in Brazil and Colombia [25,26,27], which is consistent with the restricted genomic diversity characteristic of this host-adapted serovar [11]. The repeated detection of similar PFGE profiles across years and chicken types is compatible with long-term circulation of a limited number of PFGE-defined lineages, but specific transmission routes, including vertical transmission through breeder flocks and horizontal spread between farms, cannot be resolved using PFGE alone. Previous studies have documented vector-borne transmission of SG by *D. gallinae* [6,7], which provides a biologically plausible route in Korean layer farms and supports mite control as a component of on-farm intervention, but this route was not examined in the present dataset.

Taken together, the conserved PFGE structure, temporal differences in resistance phenotype, and the higher proportion of isolates carrying longer SpvB PPL variants in the later period suggest that a PFGE-defined SG population of limited diversity has been repeatedly detected in Korean poultry flocks during most of the study period, in a setting characterized by continued vaccination and antimicrobial use. The number of isolates submitted for diagnosis has declined markedly in recent years, which is consistent with the national decline in FT occurrence. This decline has occurred alongside sustained SG9R vaccination and improved biosecurity in Korean poultry farms, but changes in diagnostic submission practices may also have contributed and the relative contribution of each factor was not assessed in this study. However, this decline does not preclude re-emergence given the environmental persistence of the organism, its transmission by *D. gallinae* [7], and the continued detection of SG9R-like field strains in Korea and elsewhere [5,8]. The isolates characterized here therefore document the phenotypic and genetic state of the Korean SG population as the disease receded and provide a reference against which any strains recovered in the future can be compared.

This study has several limitations. First, all isolates were obtained from clinical diagnostic submissions rather than from structured epidemiological sampling, and they therefore do not represent the true prevalence of SG or of antimicrobial resistance in the Korean poultry population. Submission depends on the occurrence of clinical disease and on the decisions of farmers and veterinarians, and changes in these practices over time may have influenced the composition of the collection. Second, sampling was uneven over the 25-year period. Annual isolate numbers ranged from 1 to 12, no SG was isolated in 2021–2023, and only one isolate was recovered in each of 2024–2026. This pattern is consistent with the national decline in FT, but the small number of recent isolates reduces the precision of estimates for the later years and limits the detection of rare phenotypes. The period comparisons therefore describe differences between two broad phases and should not be interpreted as a smooth longitudinal trend. Third, the chicken-type composition differed between periods. Although the main associations persisted after adjustment, the estimates were imprecise, and residual confounding by farm, region, or production system cannot be excluded. The period associations should therefore be interpreted as temporal associations within this diagnostic isolate collection rather than as population-level changes. Fourth, resistance mechanisms were not characterized at the molecular level, and no functional or infection experiments were performed on the SpvB variants. Finally, PFGE has limited discriminatory power for this highly clonal serovar. Identical or closely related PFGE profiles do not necessarily indicate membership of the same genomic lineage, and confirmation of recent common ancestry, long-term clone persistence, or specific transmission events would require higher-resolution methods such as whole-genome sequencing (WGS) with core-genome single-nucleotide polymorphism analysis, combined with epidemiological information.

## 5. Conclusions

Over a 25-year period, MDR was detected in 52.2% of the Korean SG isolates examined, with no significant difference between the two periods, but resistance profiles were simpler, and resistance to AMP, TET, and NAL was significantly lower in 2011–2026. All isolates remained susceptible to extended-spectrum cephalosporins and AZI, indicating that these agents have not yet been compromised in the SG isolates examined in this study. Longer PPL variants were more frequent in the later period, and the 21-proline variant was detected only in 2011–2026, a change whose implications for virulence warrant experimental evaluation. PFGE analysis showed that two dominant clusters were detected across years and chicken types, which is compatible with the long-term circulation of a limited number of PFGE-defined lineages within the Korean poultry industry. These findings support susceptibility-based selection and prudent use of antimicrobials for FT, and continued monitoring of field strains alongside SG9R vaccination, but they do not by themselves provide evidence for changes to vaccine policy. Future surveillance should prioritize WGS-based monitoring of resistance determinants, virulence markers, and lineage relatedness, integrated with epidemiological data.

## Figures and Tables

**Table 1:** Distribution of *Salmonella* Gallinarum isolates obtained from diseased chickens in South Korea between 2002 and 2026, by year of isolation and chicken type.

Year	No. of Isolates					
	Broiler	Layer	Broiler Breeder	Korean Native Chicken	Baeksemi	Total
2002	3	7	1	1	0	12
2003	2	3	0	0	1	6
2004	4	3	0	0	0	7
2005	3	1	0	0	0	4
2006	0	1	2	0	0	3
2007	0	1	1	1	0	3
2008	2	2	0	1	1	6
2009	4	6	0	0	0	10
2010	3	4	4	0	0	11
2011	0	2	0	3	6	11
2012	0	4	2	1	0	7
2013	0	7	0	0	0	7
2014	0	5	0	0	0	5
2015	6	1	0	0	0	7
2016	1	4	0	3	0	8
2017	0	5	0	1	0	6
2018	1	6	2	2	0	11
2019	0	3	0	2	0	5
2020	0	3	0	1	0	4
2024	0	1	0	0	0	1
2025	0	0	0	0	1	1
2026	0	1	0	0	0	1
Total	29	70	12	16	9	136

No *Salmonella* Gallinarum was isolated between 2021 and 2023.

**Table 2:** Antimicrobial resistance of *Salmonella* Gallinarum isolates recovered from diseased chickens in South Korea between 2002 and 2026.

Antimicrobial Agents	Range Tested (µg/mL)	Breakpoints (µg/mL)	Comparison of Resistance Rates of Isolates of						*p* Values	Total		
			2002–2010 (*n* = 62)			2011–2026 (*n* = 74)						
			MIC_50_	MIC_90_	% Resist. (*n*)	MIC_50_	MIC_90_	% Resist. (*n*)		MIC_50_	MIC_90_	% Resist. (*n* = 136)
Aminoglycoside												
Gentamicin	0.25–16	≥8	8	16	56.5 (35)	8	>16	54.1 (40)	0.7795 (0.7795)	8	>16	55.1 (75)
Streptomycin	2–64	≥32 ^a^	64	>64	77.4 (48)	>64	>64	87.8 (65)	0.1065 (0.1774)	64	>64	83.1 (113)
Aminopenicillin												
Ampicillin	1–32	≥32	2	>32	19.4 (12)	2	2	0	<0.0001 (0.0007)	2	16	8.8 (12)
β-Lactam/β-lactamase inhibitor												
Amoxicillin/clavulanic acid	1/0.5–32/16	≥32/16	≤1/0.5	8/4	0	≤1/0.5	2/1	0	ND	≤1/0.5	4/2	0
Cephamycin												
Cefoxitin	0.5–32	≥32	8	16	1.6 (1)	8	16	0	0.4559 (0.5699)	8	16	0.7 (1)
Cephalosporin III												
Ceftiofur	0.12–8	≥8 ^a^	1	1	0	1	1	0	ND	1	1	0
Ceftriaxone	0.25–64	≥4	≤0.25	≤0.25	0	≤0.25	≤0.25	0	ND	≤0.25	≤0.25	0
Quinolone												
Nalidixic acid	0.5–32	≥32	>32	>32	100.0 (62)	>32	>32	87.8 (65)	0.0039 (0.0130)	>32	>32	93.4 (127)
Ciprofloxacin	0.015–4	≥1	1	2	79.0 (49)	1	2	64.9 (48)	0.0688 (0.1721)	1	2	71.3 (97)
Folate pathway inhibitors												
Trimethoprim/sulfamethoxazole	0.12/2.38–4/76	≥4/76	0.5/9.5	1/19	1.6 (1)	0.25/4.75	0.5/9.5	0	0.4559 (0.5699)	0.25/4.75	0.5/9.5	0.7 (1)
Sulfisoxazole	16–512	≥512	>512	>512	53.2 (33)	256	>512	50.0 (37)	0.7077 (0.7795)	512	>512	51.5 (70)
Phenicols												
Chloramphenicol	2–32	≥32	8	8	4.8 (3)	8	8	0	0.0922 (0.1774)	8	8	2.2 (3)
Tetracyclines												
Tetracycline	4–32	≥16	≤4	>32	14.5 (9)	≤4	≤4	0	0.0006 (0.0030)	≤4	≤4	6.6 (9)
Macrolides												
Azithromycin	0.12–16	≥32 ^a^	2	2	0	2	4	0	ND	2	4	0
MDR isolates					54.8 (34)			50.0 (37)	0.5737			52.2 (71)

^a^ NARMS interpretive criteria [31]. All other breakpoints are from CLSI M100, 33rd ed. (2023) [30]. Values in parentheses in the *p* value column are Benjamini–Hochberg-adjusted *p* values across the 10 agents with at least one resistant isolate. The class headings in the first column correspond to the 10 antimicrobial classes used for MDR classification (Table S2). MIC_50_/MIC_90_, minimum inhibitory concentration required to inhibit 50%/90% of isolates; % Resist., percentage of resistant isolates; ND, not determined (no resistant isolates in either period). MDR, resistance to at least one agent in three or more of these classes.

**Table 3:** Antimicrobial resistance patterns of *Salmonella* Gallinarum isolates recovered from diseased chickens in South Korea between 2002 and 2026.

Resistance Patterns	No. of Classes	No. of Antimicrobials	% Resist. (*n*)		
			2002–2010 (*n* = 62)	2011–2026 (*n* = 74)	Total (*n* = 136)
AMP-CHL-CIP-GEN-NAL-STR-FIS-TET	6	8	1.6 (1)	0	0.7 (1)
FOX-CIP-GEN-NAL-STR-FIS-TET-SXT	5	8	1.6 (1)	0	0.7 (1)
AMP-CIP-GEN-NAL-STR-FIS-TET	5	7	8.1 (5)	0	3.7 (5)
AMP-CIP-GEN-NAL-STR-FIS	4	6	4.8 (3)	0	2.2 (3)
CHL-CIP-GEN-NAL-STR-FIS	4	6	1.6 (1)	0	0.7 (1)
CIP-GEN-NAL-STR-FIS-TET	4	6	1.6 (1)	0	0.7 (1)
AMP-CIP-GEN-NAL-FIS	4	5	1.6 (1)	0	0.7 (1)
CIP-GEN-NAL-STR-FIS	3	5	17.7 (11)	31.1 (23)	25.0 (34)
AMP-CIP-NAL-TET	3	4	1.6 (1)	0	0.7 (1)
CIP-NAL-STR-FIS	3	4	0	4.1 (3)	2.2 (3)
GEN-NAL-STR-FIS	3	4	14.5 (9)	14.9 (11)	14.7 (20)
CIP-GEN-NAL-STR	2	4	1.6 (1)	4.1 (3)	2.9 (4)
AMP-CIP-NAL	2	3	1.6 (1)	0	0.7 (1)
CHL-CIP-NAL	2	3	1.6 (1)	0	0.7 (1)
CIP-GEN-NAL	2	3	1.6 (1)	2.7 (2)	2.2 (3)
CIP-NAL-STR	2	3	19.4 (12)	16.2 (12)	17.6 (24)
GEN-NAL-STR	2	3	0	1.4 (1)	0.7 (1)
NAL-STR	2	2	4.8 (3)	5.4 (4)	5.1 (7)
CIP-NAL	1	2	12.9 (8)	6.8 (5)	9.6 (13)
NAL	1	1	1.6 (1)	1.4 (1)	1.5 (2)
STR	1	1	0	10.8 (8)	5.9 (8)
Pansusceptibility	0	0	0	1.4 (1)	0.7 (1)

Pansusceptibility indicates susceptibility to all antimicrobial agents tested. No. of classes, number of antimicrobial classes used for MDR classification (Table S2), in which CIP and NAL, GEN and STR, FIS and SXT, and XNL and CRO were each counted as one class. % Resist., percentage of resistant isolates; AMP, ampicillin; CHL, chloramphenicol; CIP, ciprofloxacin; FIS, sulfisoxazole; FOX, cefoxitin; GEN, gentamicin; NAL, nalidixic acid; STR, streptomycin; SXT, trimethoprim/sulfamethoxazole; TET, tetracycline.

**Table 4:** Distribution of SpvB PPL lengths among *Salmonella* Gallinarum isolates recovered from diseased chickens in South Korea between 2002 and 2026.

PPL Length (No. of Prolines)	Comparison of PPL Length of Isolates Between Two Periods		Total (*n* = 136)	*p* Values
	2002–2010 (*n* = 62)	2011–2026 (*n* = 74)		
13	17.7 (11)	21.6 (16)	19.9 (27)	0.0161 *
15	75.8 (47)	52.7 (39)	63.2 (86)	
17	4.8 (3)	9.5 (7)	7.4 (10)	
19	1.6 (1)	12.2 (9)	7.4 (10)	
21	0	4.1 (3)	2.2 (3)	
≥17	6.5 (4)	25.7 (19)	16.9 (23)	0.0028 **
PPL length	14.8 ± 1.1	15.5 ± 2.1	15.2 ± 1.7	

Values are presented as % (n) or mean ± standard deviation. * The distribution of PPL lengths was compared between the two periods using the Fisher–Freeman–Halton exact test, and the single *p* value applies to the whole distribution. This was regarded as the primary test for PPL length. ** Fisher’s exact test comparing isolates carrying ≥17 prolines with those carrying <17 prolines (exploratory, post hoc analysis; *p* = 0.0028). OR for 2011–2026 versus 2002–2010, 4.96 (95% CI 1.52–21.29). Both tests were two-sided. PPL, polyproline linker.

## Data Availability

The original contributions presented in this study are included in the article/Supplementary Material. Further inquiries can be directed to the corresponding author.

## Supplementary Materials

The following supporting information can be downloaded at: https://www.mdpi.com/article/10.3390/vetsci13101089/s1, Table S1: Isolate-level data for the 136 *Salmonella* Gallinarum isolates recovered from diseased chickens in South Korea, 2002–2026; Table S2: Assignment of antimicrobial agents to classes used for MDR classification, resistance breakpoints, and interpretive sources; Table S3: Analyses of the associations between study period and resistance or PPL length, adjusted for chicken type, and sensitivity analyses; Table S4: Summary of PFGE clusters (≥90% similarity) by years of isolation, chicken type, PPL length, and MDR status.

## Abbreviations

The following abbreviations are used in this manuscript:

AMP	ampicillin
aOR	adjusted odds ratio
AUG	amoxicillin/clavulanic acid
AZI	azithromycin
CHL	chloramphenicol
CI	confidence interval
CIP	ciprofloxacin
CRO	ceftriaxone
FIS	sulfisoxazole
FOX	cefoxitin
FT	fowl typhoid
GEN	gentamicin
KAHIS	Korea Animal Health Integrated System
MDR	multidrug resistance
MIC	minimum inhibitory concentration
NAL	nalidixic acid
NARMS	National Antimicrobial Resistance Monitoring System
OR	odds ratio
PCR	polymerase chain reaction
PFGE	pulsed-field gel electrophoresis
PMQR	plasmid-mediated quinolone resistance
PPL	polyproline linker
QRDR	quinolone resistance-determining region
SG	*Salmonella* Gallinarum
STR	streptomycin
SXT	trimethoprim/sulfamethoxazole
TET	tetracycline
WGS	whole-genome sequencing
XNL	ceftiofur
